# Exploring Residents’ Communication Learning Process in the Workplace: A Five-Phase Model

**DOI:** 10.1371/journal.pone.0125958

**Published:** 2015-05-22

**Authors:** Valerie van den Eertwegh, Cees van der Vleuten, Renée Stalmeijer, Jan van Dalen, Albert Scherpbier, Sandra van Dulmen

**Affiliations:** 1 Skillslab, Maastricht University, Maastricht, the Netherlands; 2 Department of Educational Development and Research, Maastricht University, Maastricht, the Netherlands; 3 Faculty of Health, Medicine and Life Sciences, Maastricht University, Maastricht, the Netherlands; 4 NIVEL (Netherlands institute for health services research), Utrecht, the Netherlands; 5 Radboud University Medical Centre, Nijmegen, the Netherlands; 6 Buskerud and Vestfold University College, Drammen, Norway; World Health Organization, SWITZERLAND

## Abstract

**Context:**

Competency-based education is a resurgent paradigm in professional medical education. However, more specific knowledge is needed about the learning process of such competencies, since they consist of complex skills. We chose to focus on the competency of skilled communication and want to further explore its learning process, since it is regarded as a main competency in medical education.

**Objective:**

This study aims to explore in more detail the learning process that residents in general practice go through during workplace-based learning in order to become skilled communicators.

**Methods:**

A qualitative study was conducted in which twelve GP residents were observed during their regular consultations, and were interviewed in-depth afterwards.

**Results:**

Analysis of the data resulted in the construction of five phases and two overall conditions to describe the development towards becoming a skilled communicator: Confrontation with (un)desired behaviour or clinical outcomes was the first phase. Becoming conscious of one’s own behaviour and changing the underlying frame of reference formed the second phase. The third phase consisted of the search for alternative behaviour. In the fourth phase, personalization of the alternative behaviour had to occur, this was perceived as difficult and required much time. Finally, the fifth phase concerned full internalization of the new behaviour, which by then had become an integrated part of the residents’ clinical repertoire. Safety and cognitive & emotional space were labelled as overall conditions influencing this learning process.

**Conclusions:**

Knowledge and awareness of these five phases can be used to adjust medical working and learning environments in such a way that development of skilled medical communication can come to full fruition and its benefits are more fully reaped.

## Introduction

Any medical treatment is carried out within a context. How contextual factors contribute to the effectiveness of the therapy that is being administered, is a fascinating field of research [1]. The powerful effect of appropriate words and attitude, as part of this context effect, has been proven in many studies by now [2–6]. Skilled communication therefore has been included as a main competency in all major competency frameworks used in medical education, like CanMeds, ACGM and Good Medical Practice [7–10]. Exactly because of the generic character of communication skills, we have chosen to explore the learning process of this competency in more detail. Competencies like communication consist of complex skills and are assumed to be learned and developed most effectively in authentic professional working contexts. However when taking a closer look at the effectiveness of current communication training programs, medical students do not seem to attain professional expertise in communication [10–13]. At postgraduate level, the situation looks even more worrisome: doctors report that they are aware of the importance of adequate doctor—patient communication, however they face difficulties when trying to apply those communication skills in their actual workplace [14–17]. Continuous formal training in communication is mostly absent (in clinical specialties other than general practice) [18,19] and when it is indeed offered, effectiveness studies demonstrate moderate to little effect in the long run [20–26].

This low level of effectiveness can partly be ascribed to deficiencies in the assessment methods used: it is argued, for instance, that assessment methods do not take proper account of context [27–30]. The complexity of the learning process itself could be another explanation for the reduced effectiveness of communication skills trainings. Wouda and Van de Wiel [31] used and adapted the reflective-impulsive model of social behavior in order to account for this complexity *theoretically*. The gist of this model is that it requires lifelong deliberate practice (in and outside the workplace) to become a skilled communicator. Previous research has aimed at the identification of barriers that residents encounter during this process of deliberate practice [32]. What the process of workplace-based learning of communication skills actually looks like *in practice*, however, has not yet been investigated further.

Different generic models do exist that describe the learning process of a new skill in general terms. One example of such a generic model is Maslow’s well-known Four Stages of Learning Model [33]. This model describes four phases a person goes through when a new skill is acquired: moving from unconsciously incompetent to consciously incompetent, then to consciously competent, to finally become unconsciously competent. These generic phases, however, have not yet been applied to or further specified when it comes to the acquisition of communication skills in the workplace. As Salmon [34] puts it, skilled communication requires more than the mastery of communication skills. But what else is required exactly? The *specific process of how* a doctor acquires and develops communication skills during medical practice should therefore be investigated further to be able to adapt curricula and help doctors develop their communication and interpersonal practices more effectively [35].

In order to do so, a closer look is needed at the concept of skilled communicator. Over the past 40 years, this concept has evolved in such a way that a continuum between two stances gradually developed [36]. The first stance focusses on the description and measurement of physician communication *behaviors* and the design and testing of interventions to improve these. The second stance focusses more on the *prerequisites* to communication competencies, such as self-awareness, reflection, compassion and self-growth objectives [37]. In order to develop good communication skills, a mixture of both stances seems desirable [38–44]. Up to now, little research has specifically explored how doctors learn, develop and fine-tune this mixture of aspects during medical practice. Besides, much is assumed about the process of learning communication, but hardly anyone has used learners’ own experiences for information on the learning process.

This study therefore aims to explore and describe the learning process GP residents report to go through during medical practice when they are developing the competency of skilled communicator.

## Methods

### 2.1 Methodology

Because of the exploratory nature of our research question “How can the learning process that GP residents undergo in order to become skilled communicators be described?” we used qualitative research methodology. The research process was informed by several core principles of qualitative research like reflexivity [45], purposive sampling, and iterative data collection and analysis [46–48]. Furthermore because of our constructivist stance we chose to perform both systematic inductive and deductive data-analysis. The latter was informed by sensitizing concepts drawn from the current literature on medical communication skills learning and—teaching, as described in the introduction. We used the Consolidated criteria for reporting qualitative research (COREQ), a 32-item checklist to make sure all important aspects were reported appropriately [49].

### 2.2 Context of the study

We chose to observe and interview residents in general practice at the Maastricht University postgraduate institute for general practice. To put it briefly, these residents receive the kind of structural support in communication skills training Bombeke et al. [39] suggest in order to bridge the gap between theory and a personal style of context-sensitive patient-centered communication. In other words, the basic conditions for learning and developing communication are met among the participants in our study, enabling us to study the learning process itself in detail. See Table 1 for an overview of the three-year postgraduate training program.

**Table 1:** Short overview of the Maastricht postgraduate training for residents in general practice.

Short overview of the Maastricht postgraduate training for residents in general practice:
• Residents spend the first year in a single GP practice. During the second year, they are trained in three clinical institutions such as the nursing home, emergency room or psychiatry ward. For the whole final year they are immersed in yet another GP practice.
• Every week, residents in year 1 and year 3 attend a one-day follow-up program at the university’s family practice department. During these meetings residents receive communication skills training through the discussion of their consultations that were videotaped during practice. These sessions are facilitated by a GP and a behavioural scientist.
• In the practice settings, GP residents are supervised by GP trainers who have received special training to provide feedback and continuous individual support.
• Residents are tested for their communication skills in a summative assessment at the end of each year: they need to hand in 40 video recordings of their patient consultations, of which six are used for the assessment using a validated behavioural rating scale.

### 2.3 Data collection

Previous data, collected from seven focus group interviews among residents GP and residents surgery, revealed factors that they perceived as enabling or hindering their application of communication skills in real practice [32]. A secondary analysis on this data was used to guide the focus of the observations and set-up of the interviews in the current study.

In the next step, we used purposeful sampling [50] within an iterative process of data collection and analysis for the selection of twelve residents from general practice in order to observe and interview them during their regular consultations between April and October 2014. The selection was based on residents’ progress in training, expressed in terms of years of experience. Permission was obtained from the head of the General Practice department. First year and third year GP residents were approached during one of their weekly sessions at the department of family practice. We did not approach 2^nd^ year residents since their learning and working context differed from year 1 and 3 (the 2^nd^ years did not stay in a GP practice and they did not have to make video recordings of consultations, see Table 1). After we explained the setup of the study, residents were invited to volunteer for participation. 14 first-year and 11 third-year residents volunteered. Out of these volunteers, we randomly chose six first-year and six third-year residents. The two supervisors of the weekly sessions checked the random selection of these twelve participants to make sure that the selection covered varying levels in communication skills. Their checking did not result in replacing or changing any of the randomly chosen twelve participants.

The study was approved by the Ethics Committee of the Dutch Association for Medical Education (NVMO)(file no. 345).

The first author, VvdE, visited each resident at his or her general practice to observe randomly chosen, regular patient contacts (shadowing technique). All observations were automatically video-taped as part of the 40 video recordings the resident has to hand in at the end of the year. After one hour of observation, an in-depth interview was conducted using the technique of stimulated recall [51]. Concrete behaviours concerning communication skills witnessed during the observation, formed an entry to the interview by asking how the resident had learned that particular behaviour or skill. In case the resident did not recall what the interviewer meant, the videotape could be used to indicate concrete behaviours or skills and thereby stimulate recall.

The aim was to get an idea of the learning process going on within that resident during workplace-based learning when he or she was applying or trying to apply a communication skill. The first questions used to probe this process were: When you think back about the past hour of consultations, what communication skill did you consciously apply? How did you learn that particular skill? How did you manage to integrate that skill and make it authentic? Which mechanism(s)/ cognitive processes/ thoughts played a role in this (what did you think when doing or saying X)?

One of the first interviews was witnessed by the second author in order to make explicit and share the perspective and effect of the interviewer on the data collection process, according to the principles of reflexivity [45]. Written informed consent was obtained from each participant.

Because we used the method of iteration [47,48] we subjected new data to analysis as soon as they were collected in order to refine subsequent data collection. This enabled us to confirm, elaborate, or refute the main insights in a stepwise manner. The final interviews revealed no new information anymore and it was decided to complete data collection after twelve interviews since saturation was achieved [50].

### 2.4 Analysis

With the participants’ written consent, all interviews were audiotaped and transcribed verbatim. Each participant was asked for consent with the content of the transcripts. VvdE and JvD independently analysed and coded the transcripts. Using principles of constant comparison [46] and the immersion/ crystallisation analysis style as described by Miller and Crabtree [52] the researchers analysed the coded data, clustered these into coherent categories and refined the categories by systematically checking them against incoming data. This analysis involved decontextualisation (to allow parts of the data to be lifted out and investigated more closely) and recontextualisation (to make sure the categories still agreed with the context from which they were collected) of the data. During this process VvdE and JvD took notice of and shared the effect of their own frame of reference (reflexivity) on the process of analysis [45]. Final consensus was reached about the construction of five specific phases and two overall conditions to describe the learning process. All co-authors discussed and agreed on the five main phases and two conditions. This procedure, known as researcher triangulation, ensures an objective or fair image of the data.

## Results

Analysis of the data resulted in the construction and description of five phases that characterized the learning process of communication skills for our particular group of GP residents during medical practice (see Table 2). All participants gave examples of the fact that their learning process was influenced by the safety of their relationship with their supervisor and by the amount of cognitive and emotional space they experienced during the workplace-based learning of their communication skills. We captured these influencing factors and labeled them as two overall conditions for learning: cognitive & emotional space and safety (see Table 2). This paragraph will provide a description of each of the phases, illustrated by quotes drawn from the interviews, and will end with a brief description of the two overall conditions. Table 2 gives an overview of the 5 phases and 2 overall conditions.

**Table 2:** The five phases and two overall conditions in GP residents’ learning process of communication skills.

Cognitive and Emotional space				
Phase 1	Phase 2	Phase 3	Phase 4	Phase 5
Confrontation with the effect of a behavior	Becoming conscious of own behavior	Searching and receiving alternative behavior	Personalization of new behavior	Internalization and clinical integration
**Safety**				

### 3.1 Phase 1: Confrontation with the effect of a behavior

The very first step in the learning process of communication skills appeared to be confrontation with a positive or negative effect of one’s own behaviour. This confrontation could occur during diverse work or personal situations; such as a very effective or ineffective consultation, by watching and discussing video recordings of consultations, or in the form of feedback during real observations. Life events, such as getting married, getting children, or illness among themselves or their family, formed a fourth important source of confrontation. In all cases, the unexpected effect or a feeling of (dis)satisfaction with the situation was the trigger to change one’s own view or way of doing, as can be read in the quotes in Table 3.

**Table 3:** Quotes accompanying phase 1.

- “I didn’t check the auxiliary question and at the end of the consultation I discovered that the parents did not necessarily require a photo too. This negative outcome really bothered me. My supervisor had told me before about the importance of consciously asking questions, but this confrontation made me all of a sudden realize what he meant. It made me become aware of my own blind spot” Rgp 7
- “When my father was in intensive care and I spoke to a resident on duty, I realized how much my emotions gained the upper hand and for the first time I was being confronted with the feeling of how it is to be a patient (‘s relative). This experience really changed my way of looking at communication. The importance I attach to good communication has really increased due to this confrontation.” Rgp 11

### 3.2 Phase 2: Becoming conscious of own behavior

The second step in the learning process consisted of reflecting upon and becoming conscious of one’s own particular behavior and its underlying mechanism or reason. Understanding the need to change that particular behavior also appeared crucial in this phase. A typical consequence of becoming conscious of and reflecting upon one’s own behavior was the automatic change in the resident’s frame of reference. These made him or her look at a situation or interaction from a different angle, and see his or her own share in the process.

Residents mentioned that life events had the deepest impact and brought about the most profound changes in their frames of reference. These changes almost always led to an improvement of their ability to empathize with patients and made them more open to other points of view. The quotes in Table 4 illustrate this second phase.

**Table 4:** Quotes accompanying phase 2.

- “My husband’s sudden illness and a personal life event have really influenced my frame of reference. I became more aware of many subtle aspects in the non-verbal communication related to patients’ fears and uncertainties. Due to this increased awareness, I now communicate with patients differently: I pay much more attention to the patient’s possible concerns. This increased awareness is a consequence of my own confrontation with suffering and illness” Rgp 10
- “I have become conscious of the three layers that are inherent to a consultation: first there is medical knowledge; on top of that comes the layer to make it a pleasant and smooth consultation, which is followed by a layer of my own feelings during a consultation that I should put to effective use. This way of looking has really influenced my communication.” Rgp 2

### 3.3 Phase 3: Searching and receiving alternative behavior

As a third step in the learning process it appeared necessary to guide the resident in making the transition from *awareness* of the need to change to *application* of new behavior in real practice. Key in this was the availability of concrete alternative behavior. Residents used many sources to search or receive suggestions for alternative behavior: they discussed feedback with the behavioral scientist /clinical trainer until they had crystalized out concrete examples of sentences they could try out in practice; they watched video recordings of peers, or observed their supervisor as role model during real observations and distilled practical alternative sentences or ways of behaving; some of them read books on communication containing tips or alternative behavior; and most of them also discussed issues in private sphere with their partner, peers, friends, family, or relatives to receive alternative tips or ideas. The range of practical alternatives did not feel as “theirs” yet and needed to be tried out, as explained in the next phase. Table 5 offers some quotes to illustrate phase 3.

**Table 5:** Quotes accompanying phase 3.

- “Actually there is a big difference between acknowledging the need to change and knowing how to change. I really need concrete tips or alternatives to make it practical and to know what I can do differently.” Rgp 1
- “Once I have become aware of a specific behaviour (and I realize that I want/ need to change it) it becomes important to have concrete alternatives ….in order to get such concrete alternatives I often talk about it with other people among my acquaintances. A friend of mine, for instance, is a psychologist, and with her I exchange tips and experiences. Her tips are always very concrete and I can try it out during new consultations to see what fits me best” Rgp 3
- “Because we discuss it extensively at the follow-up day and I then also get to see and hear other people’s reactions through the discussion of our videotaped consultations, I often return with a few alternative phrases that I can try out in my own practice to see which one works best for me.” Rgp 6

### 3.4 Phase 4: Personalization

In this phase the resident searched for situations in his or her daily practice in which the new or alternative behaviors could be tried out. Typical in this phase were the many attempts needed to find out which alternatives suited the resident best. To achieve this, learning by trial and error turned out to be the most important approach in which, moreover, the resident’s own feeling was leading. In this experiential phase, most residents reported that they had to consciously step outside of their comfort zone. Residents therefore perceived this as difficult, needing much time. The patient’s reaction or response formed an important source of information: when the patient’s reaction was negative, the resident either stopped or tried alternative ways of formulating or behaving, depending on his or her level of self-confidence and the context of the consultation. When the patient’s reaction was positive, this reassured the resident and encouraged him to try out the new behavior also in other situations. This fine-tuning and personalization was only tried out when a consultation was suitable, since each consultation differed in context and with regard to the personality of the patient.

An important characteristic all residents reported was to “be able to be myself during a consultation”, in other words “to stay authentic”. The more a new behavior was in line with their personality the easier it was to find “the right way of formulating”. In other words: “the right way of formulating” appeared to be that formulation that suited their own personality. To enlarge their repertoire, they repeatedly turned back to phase three until they found “a way of formulating” that felt right.

When a new skill didn’t produce a desirable effect or didn’t feel right after a few trial sessions, most residents abandoned the new skill, because staying authentic was more important.

A final characteristic of this phase was the long time it took to personalize a new skill. Repetition and rehearsal turned out to be crucial. See Table 6 for some quotes.

**Table 6:** Quotes accompanying phase 4.

“At first I did things in order to pass a test, but now I first consciously decide whether or not I am in harmony with it. In the process of acquiring communication skills, I am consciously trying to stay true to myself. In the past period I have gained a certain level of selfconfidence, which gives me the COURAGE to stay true to myself. This implies that I do not randomly incorporate all feedback I get during our meetings at the institute. Instead I discuss or search until I get a few alternatives that match my personality and then I go and try out these alternatives in real consultations and I continue just as long until one of them feels natural or authentic.” Rgp 8
“What matters the most to me is that a new tip or behaviour feels in line with who I am as a person; only then will I continue to try it out, make it authentic and part of my clinical repertoire. My frame of reference determines whether or not something fits me and how it makes me feel. My feelings are my most important counsellor in trying out new communication skills.” Rgp 9

### 3.5 Phase 5: Internalization and integration

When the phase of personalization had continued for a sufficient amount of time, residents reported the new skill to have become part of their regular repertoire and they applied it subconsciously. This final phase was entered when they felt the new skill had become an integrated part of themselves (internalization) and of their way of doing a consultation (integration). Characteristic of the *internalization* was the fact that the resident reported the feeling of having become more mature as a person. A natural result of this feeling was the fact that all participants reported that communication skills development and personal growth could not be seen as two independent things, but instead are integrated and should be treated as such.

Characteristic of the *integration* was the flexibility with which the skill was used: the skill was no longer used in each consultation as a goal in itself to discover its potential added value, but instead the skill was applied only when it seemed clinically or medically relevant within a specific consultation. In other words, the skill had been integrated in the clinical repertoire of the resident. Table 7 provides some quotes to illustrate phase 5.

**Table 7:** Quotes accompanying phase 5.

-“There’s a clear difference between the trial and error phase where I consciously try out and apply a specific skill to make myself familiar with it and make it personal or authentic; and the next step when the skill has become fully integrated. In this latter step I no longer apply the skill to each consultation, but only when necessary. By this I mean to say that in the end I really apply it autonomously and I SENSE it when it is needed.” Rgp 5
-“Personal growth and communication skill development run parallel to each other and cannot be separated. This is precisely because you develop as a human being, also in other spheres. You cannot regard these things separately. A new behaviour feels authentic and not just a trick only when it has become an integrated part of whom you are as a person.” Rgp 4

### 3.6 Two overall conditions influencing the learning process

All participants gave examples of factors that influenced their learning process. We clustered these factors and labelled them as two overall conditions influencing the learning process.

First of all, a relationship with their supervisors in which the residents felt safe was perceived as important. This safety was experienced when supervisors gave much personal attention, when issues were discussed in a non-judging manner and when the relationship was perceived as equal (as opposed to hierarchical) and reciprocal in learning. This safety was perceived important because residents felt vulnerable when they had to let go of old behavior and try out new behavior. It takes some courage to try out new behavior in real practice. Being assessed on their communication behavior was perceived as inconsistent with this need for a safe learning environment. The quotes in Table 8 illustrate this.

**Table 8:** Quotes accompanying overall condition 1: safety.

- “I can search and try out new behaviour because I’m in a controlled environment where I’m allowed to learn and try out. This feeling of safety is very important to me” Rgp1
- “What I really like about the conversations I have with my supervisor, is that we exchange our experiences and this feels like a reciprocal relationship in which I feel safe enough to be myself and develop myself”. Rgp 12
- “A safe climate is important to me when I am trying out new communication skills. And I feel unsafe when I am being judged for trying out. So assessment really works counterproductive”.Rg p 4

A second overall condition all residents gave examples of was labelled as “having cognitive and emotional space”. Having cognitive space means that residents felt confident with regard to the medical content or were no longer preoccupied with the medical content and this enabled them to have enough space to focus on the patient and his or her non-verbal signals. Five third year participants mentioned explicitly that their increased knowledge and confidence with regard to medical content made it possible to focus more on fine-tuning communicational aspects.

Having emotional space means the absence of emotional stressors like little sleep, bad day, personal conflicts, feelings of uncertainty, busy schedules, not enough support from their supervisor, intensive use of computer, etc. Experiencing enough emotional space was also reported as an important condition to be really able to pay undivided attention to themselves and the interaction with their patient. See quotes in Table 9.

**Table 9:** Quotes accompanying overall condition 2: cognitive and emotional space.

- “Because I had to work for a while with patients who did not require complex medical knowledge, I had room in my head for the refinement of my communication skills.” Rgp 2
- “When I am tired or not feeling very well myself or when I am stressed because the consultations are overrunning too much, then I don’t pay attention to the (more subtle) nonverbal signs of the patient anymore and I don’t have the energy to try out new communication skills.” Rgp 3

## Discussion

### 4.1 Discussion

This study gives more insight into the process of how residents in general practice learn, develop and fine-tune their communication during medical practice. We choose to focus on communication as complex skill, because communication is one of the generic competencies in all major competency frameworks. The five phases and two overall conditions we found show what it takes to personalize, internalize and integrate a new communication skill. Maslow’s general phases (moving from unconscious incompetence to unconscious competence) are acknowledged in this regard, as might be the case for other existing *generic* models on learning, but the added value of our phases is a more detailed description of the *specific processes* that take place when workplace-based *communication learning* is concerned.

In general, the assumption that residents or doctors will learn a communication skill if you “just” give them good feedback, appears to not cover the full picture. Personalization and internalization seem crucial “extra “steps in the learning process to make the new behavior authentic and effective in the long run.

The five phases might provide an additional explanation of earlier findings [53,54], stating that longitudinal training in communication skills is more effective compared to single or isolated training moments. In light of our phases, longitudinal training might increase the chance for the learner to also complete the final two phases of personalization and internalization & integration; provided that (s)he is given the opportunity and support to practice. In a study by Eertwegh et al. [32], residents in surgery for instance attained once a year an off-site communication training of maximum one day. Back on the work floor they reported the newly acquired skills or tips to evaporate quickly, since they received no support or opportunity to practice and no follow up was given on the attended training. It might be interesting to find out whether this indeed can be ascribed to the fact that these residents were not fully able to pass through the phases of personalization, internalization and integration. If so, it would be interesting to explore and evaluate how knowledge of the five phases reported in this study can be used to influence clinical workplace-based learning programs to improve long term development of skilled medical communication. We do realize however that implementing such continuous learning in clinical working environments other than general practice, requires substantial change at the institutional/hospital level.

More generally, we advocate increased awareness among doctors of the therapeutic effect of good communication [1,55]. As Bensing [55] pointed out, it is a pity that doctors do not make more conscious use of this therapeutic effect for the benefit of increased health outcomes and reduced costs. We do underline this need and we think the five phases lend themselves well to develop and fine tune workplace-based learning programs that foster an increased awareness and active use of the therapeutic effect of good communication among residents and doctors.

#### Limitations of the study

We limited ourselves to residents general practice because they met the basic conditions for learning and developing communication skills. To increase representativeness of the findings, it would be interesting to further investigate whether residents in other specialties also recognize these five phases and overall conditions in their communication learning process.

Our sample size was limited to twelve particpants. Although this might seem a rather small sample size to draw general conclusions from, the patterns and final five phases that resulted from this sample contain important information representing underlying patterns [45,50]. Our findings should be thought of as descriptions and notions applicable within our specified setting of GP residents [45]. Future research based on larger sample sizes might further generalize our findings.

### 4.2 Practice implications

The five distinct phases and two overall conditions in the acquisition of communication skills by GP residents show what it takes to learn and develop communication skills effectively. This knowledge could inspire organizers of communication skills courses from other disciplines than general practice to pay attention to other necessary components of teaching.

The focus of the current research is on communication, since it is an important competency in all competency frameworks [7–10]. The question rises whether the way communication is learned is representative for other complex skills, for example such as professionalism, interprofessional practice or leadership skills. Further research needs to address the generality of the current model for other complex skills. If it is, then this may have serious consequences for the design of longitudinal and continuous postgraduate training programs.
